# Supplementary material for: A Self-Assembling Amphiphilic Peptide Dendrimer-Based Drug Delivery System for Cancer Therapy
Source: Pharmaceutics. 2021 Jul 17;13(7):1092. doi: 10.3390/pharmaceutics13071092 (PMC8309127; doi:10.3390/pharmaceutics13071092)
Supplement: Supplementary file 1 [file pharmaceutics-13-01092-s001.zip › pharmaceutics-1285968-supplementary.pdf]

pharmaceutics

MDPI

Supplementary Materials: A Self-Assembling Amphiphilic Peptide Dendrimer-Based Drug Delivery System for Cancer Therapy

Dandan Zhu, Huanle Zhang, Yuanzheng Huang, Baoping Lian, Chi Ma, Lili Han, Yu Chen, Shengmei Wu, Ning Li, Wenjie Zhang and Xiaoxuan Liu

1. Materials

N,N,N',N'-tetramethyl-(1H-benzotriazol-1-yl)uranium-hexafluorophosphate (HBTU). Boc-L-Lys(Boc)-OH, 1-hydroxybenzotriazole (HOBt) were purchased from GL Biochem. Ltd. (Shanghai, China). Trifluoroacetic acid (TFA), propargylamine, sodium azide (NaN_3), 1-bromooctadecane, N,N-diisopropylethylamine (DIPEA) was purchased from Energy Chemical Ltd. (Shanghai, China). Doxorubicin hydrochloride was purchased from Beijing Fengtai Hualian Co. Ltd. (Beijing, China). Cell Counting Kit-8 (CCK-8), 3-(4,5-dimethylthiazol-2-yl)-2,5-diphenyltetrazolium bromide (MTT) was purchased from Sigma-Aldrich (Merck Life Science, Shanghai, China). Petroleum ether (PE), ethyl acetate (EA), dichloromethane (CH_2Cl_2), methyl alcohol (MeOH), dimethylformamide (DMF), and tetrahydrofuran (THF) were distilled before use.

All the other reagents and solvents were used without any further purification from commercial sources.

2. Synthesis and characterization of AmPDs

2.1. Synthesis and characterization of hydrophobic alkyl chain

The synthetic protocol of hydrophobic alkyl chain was optimized according to the reported literature [1-4].

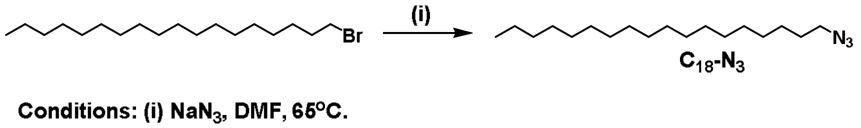

Scheme S1. Synthesis and conditions of hydrophobic alkyl chain

C_{18}-N_3: NaN_3 (171 mg, 2.62 mmol) and 1-Bromooctadecane (350 mg, 1.05 mmol) were dissolved in anhydrous DMF (4.00 mL) under nitrogen atmosphere. The mixture was stirred for 16 h at 65 °C. The mixture was diluted with ethyl acetate (50.0 mL) and then washed with pure H_2O (25.0 mL × 3) and saturated NaCl aqueous solution (30.0 mL). After that, the solution was dried with Na_2SO_4 for 2 h. The solvent was evaporated, yielding **C_{18}-N_3** (276 mg, 89%) as colorless oil. ^1H NMR (300 MHz, $CDCl_3$) δ 3.25 (t, *J* = 7.0 Hz, 2H), 1.68 – 1.54 (m, 2H), 1.48 – 1.14 (m, 30H), 0.88 (t, *J* = 6.6 Hz, 3H).

2.2. Synthesis and characterization of peptide dendrons

The synthetic protocol of peptide dendrons was optimized according to the reported literature [5,6].

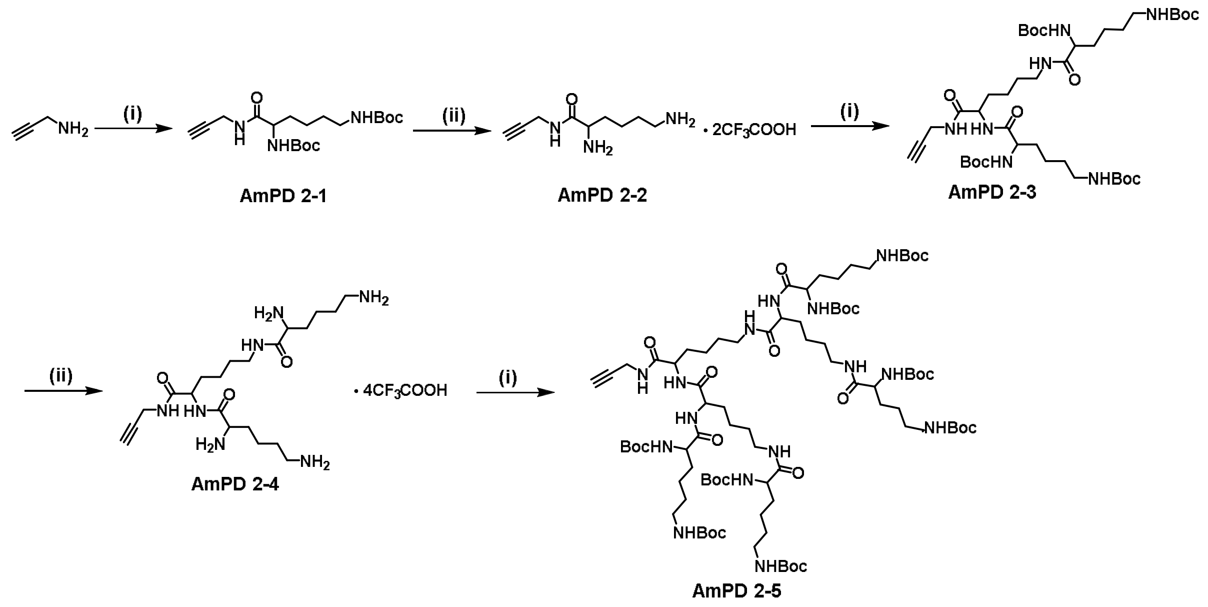

Conditions:

(i) Boc-L-Lys(Boc)-OH, HBTU, HOBt, DIPEA, DMF, 30°C;

(ii) TFA/DCM, RT;

Scheme S2. Synthesis and conditions of AmPDs.

AmPD 2-1: Boc-L-Lys(Boc)-OH (2.49 g, 7.19 mmol), HBTU (3.18 g, 8.39 mmol) and HOBT (1.13 g, 8.39 mmol) and DIPEA (4.00 mL, 24.0 mmol) were dissolved in anhydrous DMF (20.0 mL) under nitrogen atmosphere and stirred at 0 °C for 30 min. A solution of propargylamine (330 mg, 5.99 mmol) in DMF (1.00 mL) was added to the above solution. The mixture was stirred under nitrogen for 24 h at 30 °C. After solvent evaporation, the mixture was dissolved in ethyl acetate (150 mL) and washed with saturated $NaHCO_3$ aqueous solution (50 mL × 3), 1 N HCl aqueous solution (50.0 mL × 3), and saturated NaCl aqueous solution (50.0 mL × 3). The solution was dried with Na_2SO_4 for 2 h. After solvent evaporation, the residue was purified by silica gel column chromatography with Petroleum ether (PE) / ethyl acetate (EA) (1/1), yielding AmPD 2-1 (2.00 g, 87%) as white solid. ^{1}H NMR (300 MHz, $CDCl_3$) δ 6.53 (br, 1H), 5.12 (br, 1H), 4.60 (br, 1H), 4.15 – 3.94 (m, 3H), 3.10 (q, *J* = 6.6 Hz, 2H), 2.21 (t, *J* = 3.0 Hz, 1H), 1.94 – 1.75 (m, 2H), 1.68 – 1.29 (m, 22H). ESI-MS: calcd. for $C_{19}H_{33}N_3O_5$ $[M+Na]^+$406.2, found 406.3.

AmPD 2-2: AmPD 2-1 (800 mg, 2.09 mmol) was dissolved in anhydrous dichloromethane (3.10 mL), TFA (3.10 mL, 41.7 mmol) was added to the above solution under stirring at 0 °C. The reaction solution was stirred under nitrogen for 2 h at ambient temperature. After solvent evaporation, the residue was washed with anhydrous diethyl ether (20.0 mL × 3), yielding AmPD 2-2 (858 mg, 95%) as yellow oily solid. ^{1}H NMR (300 MHz, D_2O) δ 4.09 – 3.87 (m, 3H), 2.95 (t, *J* = 7.5 Hz, 2H), 2.58 (t, *J* = 2.4 Hz, 1H), 1.92 – 1.80 (m, 2H), 1.72 – 1.57 (m, 2H), 1.45 – 1.31 (m, 2H).

AmPD 2-3: Boc-L-Lys(Boc)-OH (566 mg, 1.63 mmol), HBTU (620 mg, 1.63 mmol), HOBT (221 mg, 1.63 mmol) and DIPEA (1.20 mL, 6.81 mmol) were dissolved in anhydrous DMF (3.00 mL) under nitrogen atmosphere and stirred at 0 °C for 30 min. A solution of AmPD 2-2 (280 mg, 0.68 mmol) in DMF (2.00 mL) was added to the above solution under stirring. The reaction solution was moved to 30 °C and continued to stir under nitrogen for 48 h. After solvent evaporation, the mixture was dissolved in ethyl acetate (50 mL) and washed with saturated $NaHCO_3$ aqueous solution (30.0 mL × 3), 1 N HCl aqueous solution (30.0 mL × 3), and saturated NaCl aqueous solution (30.0 mL × 3). The mixture was dried with Na_2SO_4 for 2 h. After solvent evaporation, the residue was purified by silica gel column chromatography with PE/EA (1/4), yielding AmPD 2-3 (458 mg, 80%) as white solid. ^{1}H NMR (300 MHz, DMSO-d_6) δ 8.34 (t, *J* = 4.8 Hz, 1H), 7.78 – 7.65 (m, 2H), 6.88 (d, *J* = 7.8 Hz, 1H), 6.82 – 6.28 (m, 3H), 4.21 (q, *J* = 7.8 Hz, 1H), 3.93 – 3.73 (m, 4H), 3.11 (t, *J* = 3.0 Hz,

1H), 3.05-2.81 (m, 6H), 1.68-1.04 (m, 54H). ESI-MS: calcd. for $C_{41}H_{73}N_{7}O_{11}$ $[M+Na]^{+}$862.5, found 862.7.

AmPD 2-4: AmPD 2-3 (400 mg, 0.48 mmol) was dissolved in anhydrous dichloromethane (1.40 mL), TFA (1.40 mL, 19.1 mmol) was added to the above solution under stirring at 0 °C. The reaction solution was stirred under nitrogen for 4 h at ambient temperature. After solvent evaporation, the residue was washed with anhydrous diethyl ether (20.0 mL × 3), yielding AmPD 2-4 (414 mg, 97%) as White solid. ^{1}H NMR (300 MHz, D_2O) δ 4.26 (t, *J* = 7.2 Hz, 1H), 4.10 – 3.86 (m, 4H), 3.23 (t, *J* = 6.9 Hz, 2H), 3.08-2.92 (m, 4H), 2.63 (t, *J* = 2.4 Hz, 1H), 2.01 – 1.85 (m, 4H), 1.84 – 1.65 (m, 6H), 1.63 – 1.28 (m, 8H).

AmPD 2-5: Boc-L-Lys(Boc)-OH (888 mg, 2.56 mmol), HBTU (972 mg, 2.56 mmol), HOBT (346 mg, 2.56 mmol) and DIPEA (1.50 mL, 9.15 mmol) were dissolved in anhydrous DMF (4.00 mL) under nitrogen atmosphere and stirred at 0 °C for 30 min. A solution of AmPD 2-4 (410 mg, 0.46 mmol) in DMF (3.00 mL) was added to the above solution. The mixture was stirred under nitrogen for 48 h at 30°C. After solvent evaporation, the mixture was dissolved in ethyl acetate (70 mL) and washed with saturated $NaHCO_3$ aqueous solution (40.0 mL × 3), 1 N HCl aqueous solution (40.0 mL × 3), and saturated NaCl aqueous solution (40.0 mL × 3). After solvent evaporation, the residue was purified by silica gel column chromatography with CH_2Cl_2/MeOH (20/1), yielding AmPD 2-5 (656 mg, 82%) as white solid. ^{1}H NMR (300 MHz, DMSO-d_6) δ 8.32 (s, 1H), 7.99 – 7.56 (m, 6H), 6.91 (s, 2H), 6.82 – 6.28 (m, 6H), 4.32 – 4.09 (m, 3H), 3.93 – 3.72 (m, 6H), 3.07 (t, *J* = 4.8 Hz, 1H), 3.04 – 2.81 (m, 14H), 1.73 – 1.06 (m, 114H). ESI-MS: calcd. for $C_{85}H_{153}N_{15}O_{23}$ $[M+Cl]^{-}$1787.1, found 1787.5.

2.3. Synthesis and characterization of AmPD KK$_2$

AmPD KK_2-Boc: AmPD 2-3 (142 mg, 0.169 mmol), $CuSO_4{\cdot}5H_2O$ (2.0 mg, 0.008 mmol) and NaAsc (4.0 mg, 0.017 mmol) were dissolved in anhydrous THF (2.00 mL) under nitrogen atmosphere. A solution of C_{18}-N_3 (60 mg, 0.201 mmol) in THF (2.00 mL) was added to the above solution. Then the mixture was added distilled water (1.00 mL) and stirred under nitrogen for 3 h at 50 °C. After solvent evaporation, the reaction mixture was extracted with CH_2Cl_2, washed with saturated NH_4Cl solution (30.0 mL × 3), brine (30.0 mL × 1), and then dried over Na_2SO_4. The residue was purified by silica gel column chromatography with CH_2Cl_2/MeOH (20/1), yielding AmPD KK_2-Boc (163 mg, 85%) as white solid. ^{1}H NMR (400 MHz, DMSO-d_6) δ 8.37 (t, *J* = 5.4 Hz, 1H), 7.82 (s, 1H), 7.73 (q, *J* = 7.6 Hz, 2H), 6.87 (d, *J* = 7.9 Hz, 1H), 6.81 – 6.27 (m, 3H), 4.34 – 4.15 (m, 5H), 3.92 – 3.75 (m, 2H), 3.06 – 2.79 (m, 6H), 1.81 – 1.70 (m, 2H), 1.67 – 1.02 (m, 84H), 0.85 (t, *J* = 6.2 Hz, 3H). ESI-MS: calcd. for $C_{59}H_{110}N_{10}O_{11}$ $[M+Na]^{+}$1157.8, found 1157.8.

AmPD KK_2: AmPD KK_2-Boc (200 mg, 0.18 mmol) was dissolved in anhydrous CH_2Cl_2 (0.52 mL), TFA (0.52 mL, 7.04 mmol) was added to the above solution under stirring at 0 °C. The mixture was stirred under nitrogen for 4 h at ambient temperature. After solvent evaporation, the residue was washed with anhydrous diethyl ether (20.0 mL × 3). The product was further purified by dialysis using dialysis tube of MWCO 100-500, followed by lyophilization to give AmPD KK_2 (199 mg, 95%) as white solid. ^{1}H NMR (400 MHz, $CD_3OD/CDCl_3$ = 3/2) δ 7.82 (s, 1H), 4.43 (d, *J* = 1.6 Hz, 2H), 4.39 – 4.26 (m, 3H), 3.91 (t, *J* = 6.5 Hz, 1H), 3.81 (t, *J* = 6.6 Hz, 1H), 3.29 – 3.13 (m, 2H), 3.01 – 2.86 (m, 4H), 1.97 – 1.15 (s, 50H), 0.88 (t, *J* = 6.8 Hz, 3H). ESI-MS: calcd. for $C_{39}H_{78}N_{10}O_3$ $[M+H]^{+}$735.6, found 735.9.

2.4. Synthesis and characterization of AmPD KK$_2$K$_4$

AmPD KK_2K_4-Boc: AmPD 2-5 (100 mg, 0.057 mmol), $CuSO_4{\cdot}5H_2O$ (1.0 mg, 0.003 mmol) and NaAsc (2.0 mg, 0.006 mmol) were dissolved in anhydrous THF (3.00 mL) under nitrogen atmosphere. A solution of C_{18}-N_3 (20.0 mg, 0.068 mmol) in THF (1.00 mL) was added to the above solution. Then distilled water (1.00 mL) was added to the mixture and stirred under nitrogen for 12 h at 50 °C. After solvent evaporation, the reaction mixture was extracted with CH_2Cl_2, washed with saturated NH_4Cl solution (30.0 mL × 3), brine (30.0 mL × 1), and then dried over Na_2SO_4. The residue was purified by silica gel column chromatography with CH_2Cl_2/MeOH (20/1), yielding AmPD KK_2K_4-Boc (63 mg,

54%) as white solid. ^1H NMR (300 MHz, DMSO-d_6) δ 8.36 (s, 1H), 7.95 – 7.63 (m, 7H), 6.92 (d, *J* = 9.3 Hz, 2H), 6.81 – 6.32 (m, 6H), 4.39 – 4.06 (m, 7H), 3.90 – 3.73 (m, 4H), 3.12 – 2.74 (m, 14H), 1.84 – 1.01 (m, 146H), 0.84 (t, *J* = 6.0 Hz, 3H).

AmPD KK_2K_4: AmPD KK_2K_4-Boc (84.0 mg, 0.04 mmol) was dissolved in anhydrous CH_2Cl_2 (0.74 mL), TFA (0.74 mL, 9.84 mmol) was added to the above solution under stirring at 0 °C. The reaction solution was stirred under nitrogen for 8 h at ambient temperature. After solvent evaporation, and the residue was washed with anhydrous diethyl ether (20.0 mL × 3). The product was further purified by dialysis using dialysis tube of MWCO 100-500, followed by lyophilization to give AmPD KK_2K_4 (82.0 mg, 92%) as white solid. ^1H NMR (300 MHz, $CD_3OD/CDCl_3$ = 3/2) δ 7.88 (s, 1H), 4.48 – 4.28 (m, 7H), 4.07 (t, *J* = 6.4 Hz, 2H), 3.96 (q, *J* = 6.4 Hz, 2H), 3.30 – 3.09 (m, 6H), 3.08 – 2.88 (m, 8H), 2.09 – 1.10 (m, 74H), 0.88 (t, *J* = 6.6 Hz, 3H). ESI-MS: calcd. for $C_{63}H_{126}N_{18}O_7$ $[M+H]^+$1248.0, found 1248.2.

3. Critical aggregation concentration of AmPDs nanoassemblies

Pyrene was chosen as a florescence probe to assess the CAC. The concentration of AmPDs nanoassemblies was varied from 7.8×10^{-3} to 1.0 mg/L, while the final concentration of pyrene was set as 2.5×10^{-5} M in phosphate-buffered saline (0.01 M). After sonicating for 5 min and resting at ambient temperature for 12 h, the solution was then detected using a fluorescence spectrophotometer. The pyrene fluorescence spectra were recorded with an excitation wavelength at 334 nm. Fluorescent intensity ratio (I_{373}/I_{384}) of the peak at 373 nm and 384 nm was calculated and plotted vs. the logarithm of AmPDs nanoassemblies concentration.

4. Preparation of Doxorubicin-loading nanoformulations.

Doxorubicin hydrochloride (2.5 mg) was dissolved in $CHCl_3$/MeOH = 1/3 and TEA was added (molar ratio of DOX/TEA = 1/3) to obtain hydrophobic DOX. The drug was then mixed with 4.0 mg AmPDs into 4.0 mL PBS (0.01 M) slowly. The unencapsulated DOX was removed via a dialysis bag (Molecular Weight Cut-Off (MWCO) = 1000 Da). The amount of doxorubicin encapsulated in the nanocarriers was measured using a microplate reader (Cytation 5, BioTek, Vermont, USA) with excitation wavelength at 477 nm and emission wavelength at 591 nm. Blank AmPD nanoassemblies were prepared through the same procedures without doxorubicin addition. The drug loading content (DLC) and encapsulation efficiency (EE) were calculated using the following equations:

DLC (%) = weight of drug loaded in nanoformulations/weight of nanoformulations × 100%

EE (%) = weight of drug loaded in nanoformulations/initial weight of feeding drug × 100%

5. Size distribution and Zeta potential measurements

The size distribution of AmPDs nanoassemblies and AmPDs/DOX nanoformulations was determined by DLS using NanoBrookOmni (Brookhaven, Long Island, N.Y. USA). The final concentrations of AmPDs in both the AmPDs nanoassemblies solution and AmPDs/DOX nanoformulations solution was 2.0 mg/mL.

6. In vitro drug release

The drug release from AmPDs/DOX nanoformulations was carried out in buffer (0.01 M, 200 μL, DOX equivalent of 1 mg) at different pH values (pH 7.4 or 5.0), respectively. Nanoformulations were dissolved in the buffer and transferred into dialysis bags (MWCO = 10 kDa). Then, these dialysis bags were immersed into a relevant buffer (30 mL) and kept in a shaking bed at 37 °C. At a series of sequential time points (0.5, 1, 2, 4, 6, 8, 10, 12, 24 h), 1 mL solution was taken out and the same volume fresh buffer was added subsequently. The doxorubicin concentration was measured using a microplate reader with excitation wavelength at 477 nm and emission wavelength at 591 nm. The cumulative amount of DOX released from nanoparticles was plotted against time.

7. Cell culture

Doxorubicin-sensitive cell line (MCF-7, human breast cancer cells) was purchased from the Tongpai Biotechnology Co. Ltd (Shanghai,China). Doxorubicin-resistant cell line (MCF-7R) was a gift from Dr. Hulin Jiang (China Pharmaceutical University, Nanjing, China). MCF-7 cells were maintained in DMEM (HyClone™-GE, Logan. UT. USA), supplemented with 10% Foundation™ Fetal Bovine Serum (FBS) (Gemini Bio-Products, Riverside Parkway, West Sacramento, US). MCF-7R cells were maintained in RMPI 1640 (HyClone™-GE, Logan. UT. USA), supplemented with 10% FBS. Cells were maintained at 37°C in a humidified atmosphere containing 5% CO_2.

8. Safety assessment

The LDH method was used to evaluate the membrane damage toxicity of AmPDs nanoassemblies on human breast cancer cells（MCF-7R cells and MCF-7 cells). The cells were seeded at a density of $5.0×10^3$ cells per well and incubated overnight. The cells were incubated with a series of AmPDs nanoassemblies with a concentration ranging from 0.01 to 20 μM for 24 h, with 3 auxiliary wells in each group, and then 10 μL of lysate was added to the positive control group and incubated at ambient temperature for 30 min. 100 μL of LDH working solution was added to each well, incubate for another 20 min, then 50 μL of stop solution was added, and immediately measure the absorbance at 490 nm.

Cell killing rate (%) = (the absorbance of the administration group-the absorbance of the negative control group)/(the absorbance of the positive control group-the absorbance of the negative control group) × 100%

9. In vitro anticancer activity

The anticancer activities of AmPDs/DOX nanoformulations on human breast cancer cells (MCF-7 and MCF-7R) were evaluated by MTT assays. MCF-7 and MCF-7R ($3 × 10^3$ cells per well) were seeded in 96-well plates and incubated at 37 °C overnight. The culture mediums were replaced with 100 μL fresh mediums including AmPDs/DOX nanoformulations and the DOX·HCl at a serial of doxorubicin concentrations ranging from 0.01 to 20 μg/mL. The untreated control cells were set as 100% viability. The cells were incubated for 24 h. MTT solution (0.10 mg/mL) was added to each well and incubated for another 4 h. After removing the mediums, the cells were resuspended in 200 μL DMSO solution. The absorbance of the DMSO solution was measured at 570 nm via a microplate reader.

10. In vitro cellular uptake

Flow cytometry: MCF-7R cells were seeded into 24-well plates with a density of $6.0 × 10^4$ cells per well and cultured for 24 h. Then, the culture mediums were replaced with free DOX and AmPDs/DOX nanoformulations (DOX equivalent of 2 μg/mL). The cells with no treatment were used as control. After 30 min and 2 h incubation at 37 °C, cells were harvested, washed, and resuspended with 200 μL PBS solution, then analyzed using flow cytometry. Meanwhile, the cellular uptake of free DOX and AmPDs/DOX nanoformulations at a doxorubicin concentration of 4 μg/mL were also observed.

Confocal microscopy: MCF-7R ($1.0 × 10^5$) cells were seeded into 2.0 cm confocal dishes. After 24 h of incubation, mediums containing samples (AmDPs/DOX nanoformulations or free DOX) were introduced into the system with a final doxorubicin concentration of 10 μg/mL. Cells were incubated for a certain period of times (1 h or 4 h). After the removal of mediums, cells were washed with PBS thrice, then stained with lysotracker green and Hoechst 33342. The cellular uptake of nanoassemblies and DOX were observed through two-photon confocal (Zeiss, Germany).

11. Intracellular drug accumulation

The MCF-7R ($6 × 10^4$ cells per well) were seeded in 24-well plates and incubated at 37°C overnight. The culture mediums were replaced with 500 μL fresh complete mediums containing AmPDs/DOX nanoformulations and the free DOX (DOX equivalent of 10

μg/mL), respectively, while the complete mediums were used as control. After 4 h of incubation, the mediums were replaced with fresh mediums. At different time points (0, 1, 2, 4, 8 h), cells were harvested by trypsinization, washed thrice with PBS solution, and resuspended in 200 μL PBS after centrifugation (1000 rpm, 5 min). The fluorescence intensity was measured (excitation: 480 nm, emission: 590 nm) via flow cytometer.

12. Drug penetration in tumor spheroids

Multicellular tumor spheroids were constructed with MCF-7R cells according to the reported protocol [7]. Poly(hydroxyethyl methacrylate) (45 mg) was dissolved in ethanol (30 mL, 95%), and then irradiated under UV for 12 h. Each of the cell culture flasks was coated with 3 mL of the solution, dried at 60°C, and then irradiated under UV for another 30 min to ensure sterile. Next, cell suspensions containing 500 MCF-7R cells were seeded into the cell culture flasks. The medium was changed every 2 days for 2 weeks in order to form the tumor spheroids.

To evaluate the drug penetration ability, the MCF-7R tumor spheroids were incubated with free DOX or AmPDs/DOX nanoformulations at a doxorubicin concentration of 10 μg/mL for 4 h. Then, the tumor spheroids were washed with PBS thrice. The tumor spheroids were transferred to confocal dishes (2.0 cm) and analyzed using a two-photon microscope (Carl Zeiss LSM7MP). Z-stack images were obtained by scanning the tumor spheroids layer by layer (× 10 μm thickness).

13. In vitro anticancer activity in tumor spheroids

The anticancer activities of AmPDs/DOX nanoformulations on tumor spheroids were assessed by MTT assays. The tumor spheroids were seeded in 96-well plates and incubated at 37°C for 4 days. The culture mediums were replaced with 200 μL fresh mediums including AmPDs/DOX nanoformulations and free DOX at a serial of doxorubicin concentrations ranging from 10 to 200 μg/mL. The untreated control cells were set as 100% viability. Cells were further incubated for 48 h. CCK-8 solution was added to each well and incubated for another 4 h. The absorbance of the solution was measured at 450 nm via a microplate reader.

14. Statistical tests

All data are presented as mean ± SD unless otherwise indicated. Statistical analysis was performed by one-way ANOVA or two-way ANOVA with Tukey's post-hoc test (Graphpad Prism 8.01). $p \leq 0.05$ was considered significant (*); $p \leq 0.01$ (**); $p \leq 0.001$ (***).

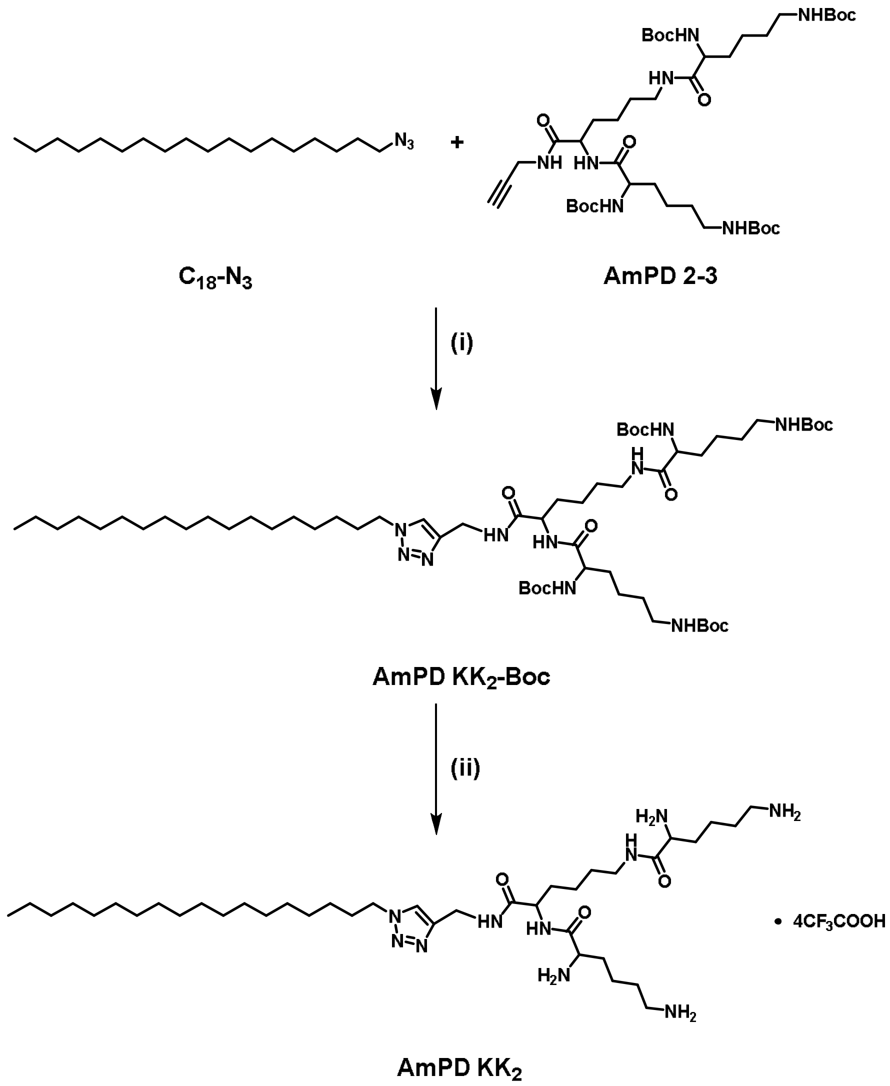

Conditions:

(i) $CuSO_4$•$5H_2O$, NaAsc, THF/H_2O, 50°C, 3h;

(ii) TFA/DCM, RT, 4h.

Scheme S3. Synthetic route of the AmPD KK_2.

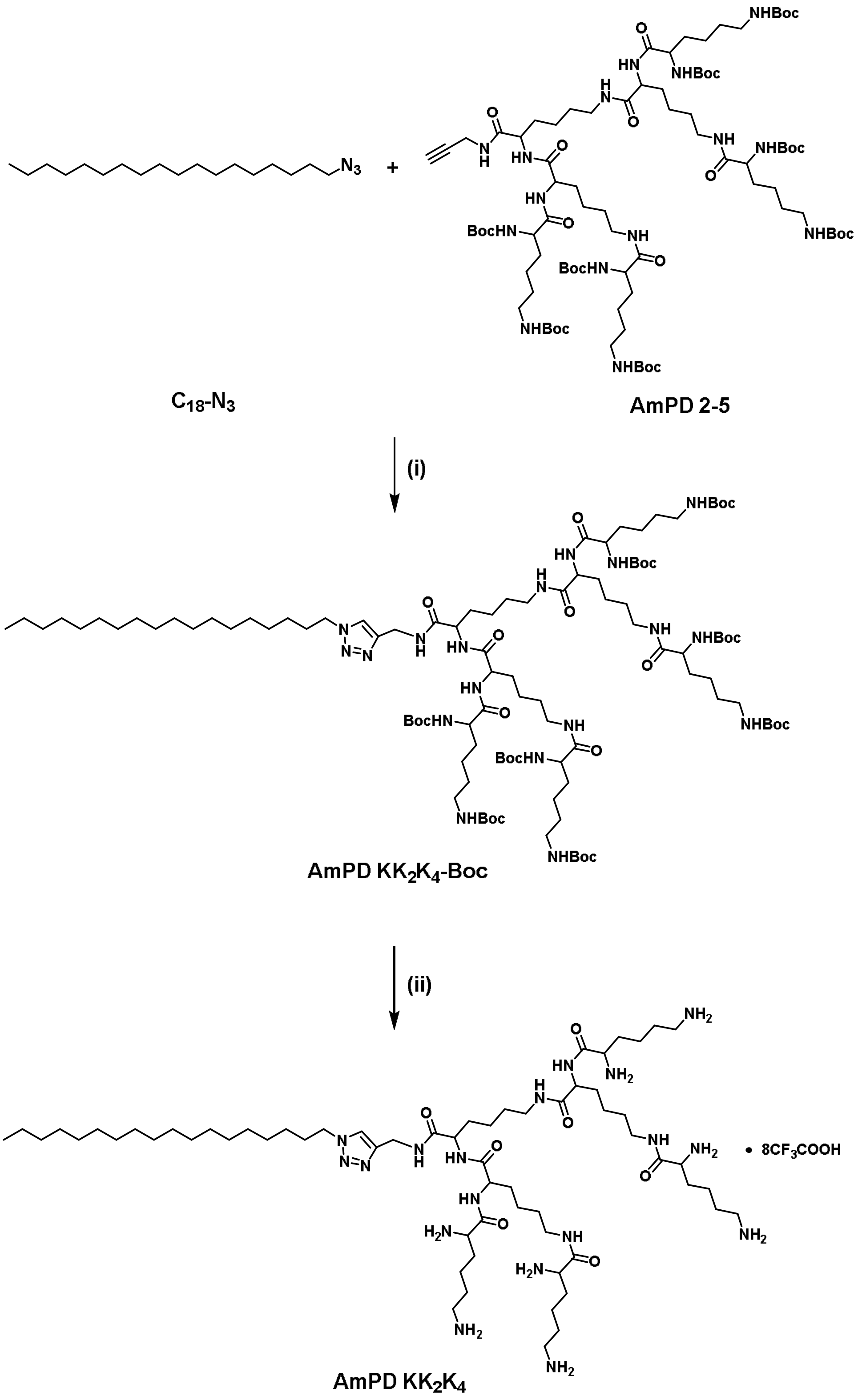

Conditions:

(i) $CuSO_4 \cdot 5H_2O$, NaAsc, THF/H_2O, 50°C, 12h;

(ii) TFA/DCM, RT, 8h.

Scheme S4. Synthetic route of the AmPD KK_2K_4.

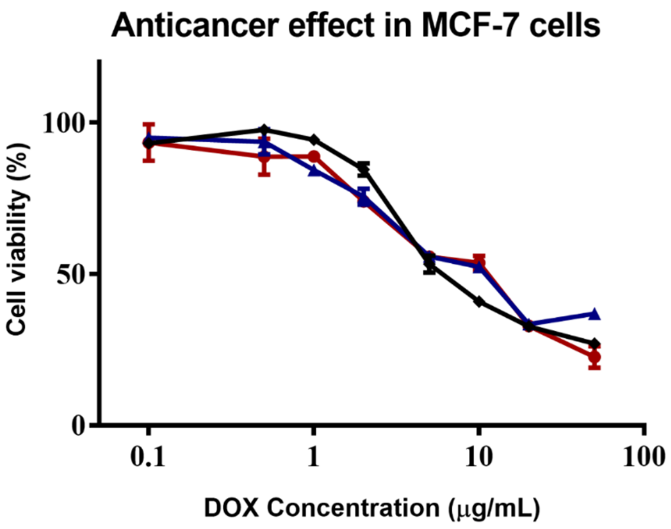

Figure S1. The antiproliferative effect of the AmPDs/DOX nanoformulations in MCF-7 cells by MTT assay (mean ± SD, n = 3).

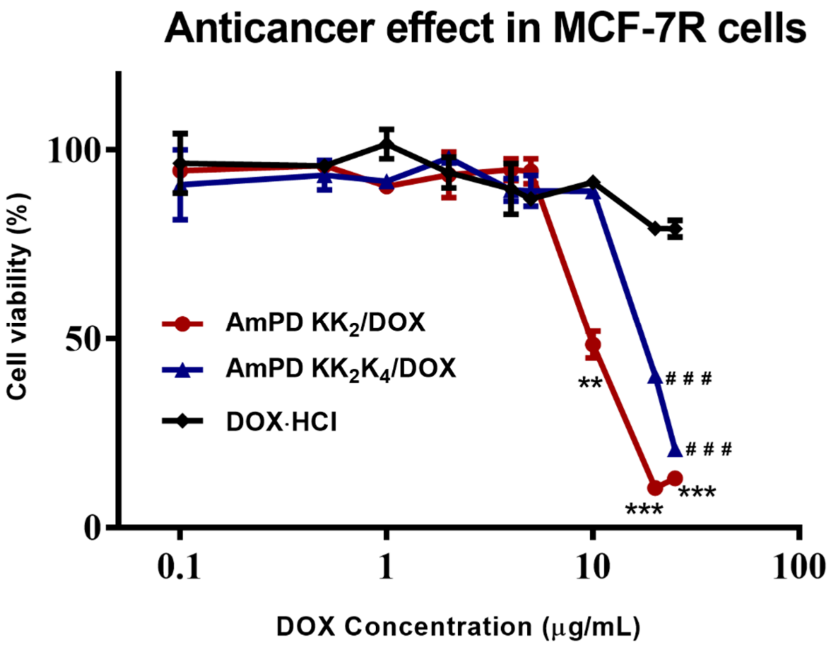

Figure S2. The antiproliferative effect of the AmPDs/DOX nanoformulations in MCF-7R cells by MTT assay (mean ± SD, n = 3, $^{###}p < 0.01$ represented AmPD KK_2K_4/DOX vs DOX, $^{**}p < 0.01$ and $^{***}p < 0.001$ represented AmPD KK_2/DOX vs DOX).

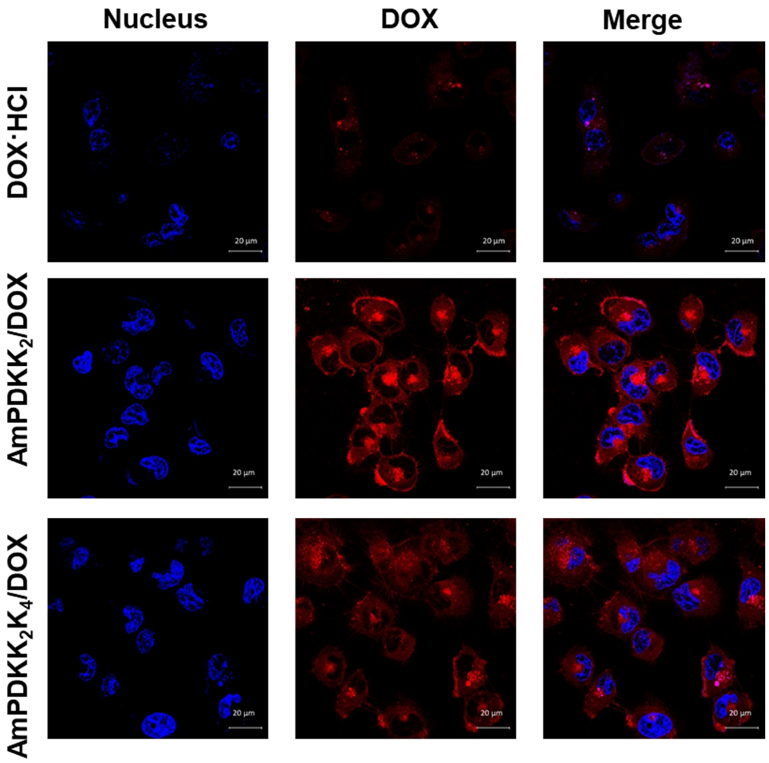

Figure S3. Confocal microscopic imaging of the cellular uptake of the AmPDs/DOX nanoformulations in MCF-7R cells. Red channel image shows DOX, and blue channel image shows the nucleus of MCF-7R cells stained by Hoechst33342.

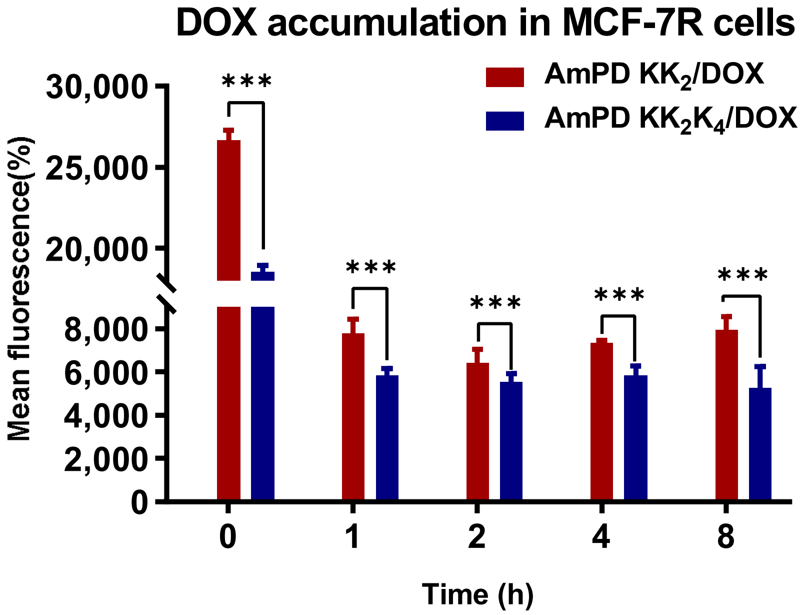

Figure S4. The intracellular accumulation of AmPDs/DOX nanoformulations in MCF-7R cells by flow cytometry analysis (mean ± SD, $n = 3$, *** $p < 0.001$).

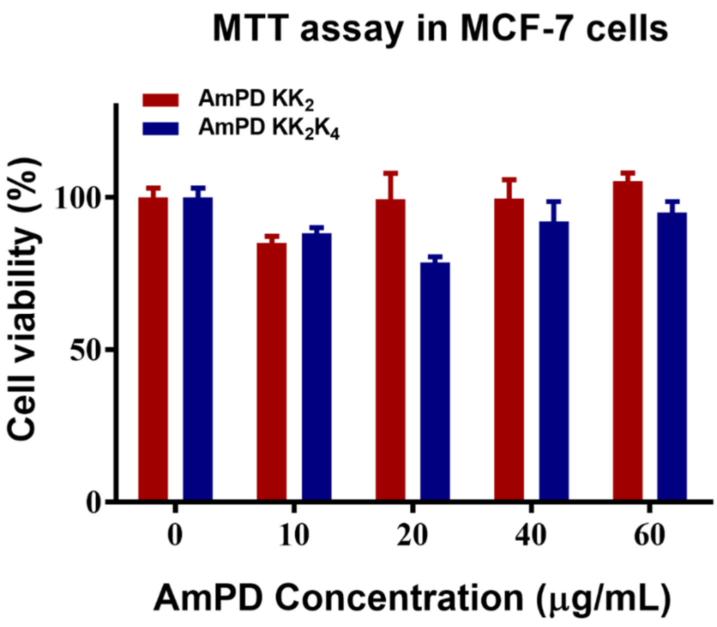

Figure S5. Metabolic toxicity of the AmPDs nanoassemblies in MCF-7 cells by MTT assay (mean ± SD, *n* = 3).

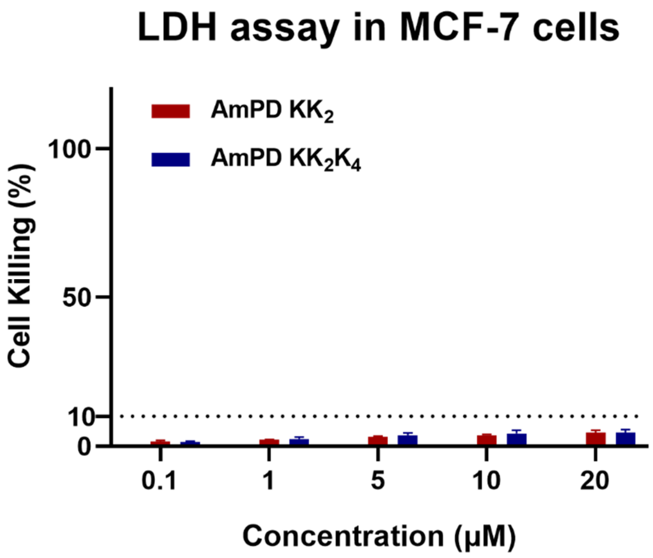

Figure S6. Membrane injury toxicity of the AmPDs nanoassemblies in MCF-7 cells by LDH assay (mean ± SD, *n* = 3).

Table S1. Size and Zeta potential of the AmPDs nanoassemblies (mean ± SD, n = 3).

Groups	DLS/nm	PDI	Zeta potential/mV
AmPD KK_2	9.4 ± 2.9	0.306 ± 0.073	12.3 ± 1.39
AmPD KK_2K_4	15 ± 3.2	0.312 ± 0.094	8.50 ± 1.41

Table S2. Data analysis of CD spectrum of AmPDs nanoassemblies by CDNN software.

Groups	Helix	Antiparallel	Parallel	Beta-turn	Randm.Coil
AmPD KK_2	6.5%	37.3%	10.6%	12.9%	32.7%
AmPD KK_2K_4	5.8%	41.2%	10.0%	13.0%	30.0%

Table S3. Size and Zeta potential of the AmPDs/DOX nanoformulations (mean ± SD, n = 3).

Groups	DLS/nm	PDI	Zeta potential/mV
AmPD KK_2/DOX	73.57 ± 4.2	0.169 ± 0.023	13.4 ± 0.99
AmPD KK_2K_4/DOX	80.38 ± 8.9	0.247 ± 0.032	11.6 ± 1.23

Table S4. The half-maximal inhibitory concentrations (IC50) of AmPDs/DOX nanoformulations in MCF-7 cells, MCF-7R cells and MCF-7R 3D-cultured tumor spheroids.

Groups	IC_{50}		
	MCF-7 cells	MCF-7R cells	MCF-7R 3D-cultured tumor spheroids
DOX·HCl	3.8 μg/mL or 6.6 μM	-	-
AmPD KK_2/DOX	2.4 μg/mL or 4.4 μM	7.0 μg/mL or 12.9 μM	48.4 μg/mL or 89.1 μM
AmPD KK_2K_4/DOX	2.6 μg/mL or 4.8 μM	26 μg/mL or 47.8 μM	138.5 μg/mL or 254.8 μM

References

1. Yu, T.; Liu, X.; Bolcato-Bellemin, A.-L.; Wang, Y.; Liu, C.; Erbacher, P.; Qu, F.; Rocchi, P.; Behr, J.-P.; Peng, L. An Amphiphilic Dendrimer for Effective Delivery of Small Interfering RNA and Gene Silencing In Vitro and In Vivo. *Angew. Chem. Int. Ed.* **2012**, *51*, 8478–8484, doi:10.1002/anie.201203920.
2. Liu, X.; Zhou, J.; Yu, T.; Chen, C.; Cheng, Q.; Sengupta, K.; Huang, Y.; Li, H.; Liu, C.; Wang, Y.; et al. Adaptive amphiphilic dendrimer-based nanoassemblies as robust and versatile siRNA delivery systems. *Angew. Chem. Int. Ed.* **2014**, *53*, 11822–11827, doi:10.1002/anie.201406764.
3. Chen, C.; Posocco, P.; Liu, X.; Cheng, Q.; Laurini, E.; Zhou, J.; Liu, C.; Wang, Y.; Tang, J.; Dal Col, V.; et al. Mastering dendrimer self-assembly for efficient siRNA delivery: from conceptual design to in vivo efficient gene silencing. *Small.* **2016**, *12*, 3667-3676.
4. Liu, X.; Wang, Y.; Chen, C.; Tintaru, A.; Cao, Y.; Liu, J.; Ziarelli, F.; Tang, J.; Guo, H.; Rosas, R.; et al. A Fluorinated Bola-Amphiphilic Dendrimer for On-Demand Delivery of siRNA, via Specific Response to Reactive Oxygen Species. *Adv. Funct. Mater.* **2016**, *26*, 8594–8603, doi:10.1002/adfm.201604192.
5. Ma, C.; Zhu, D.; Chen, Y.; Dong, Y.; Lin, W.; Li, N.; Zhang, W.; Liu, X. Amphiphilic peptide dendrimer-based nanovehicles for safe and effective siRNA delivery. *Biophys. Rep.* **2020**, *6*, 278–289, doi:10.1007/s41048-020-00120-z.
6. Luo, K.; Li, C.; Li, L.; She, W.; Wang, G.; Gu, Z. Arginine functionalized peptide dendrimers as potential gene delivery vehicles. *Biomaterials* **2012**, *33*, 4917–4927, doi:10.1016/j.biomaterials.2012.03.030.
7. Nath, S.; Devi, G.R. Three-dimensional culture systems in cancer research: Focus on tumor spheroid model. *Pharmacol. Ther.* **2016**, *163*, 94–108, doi:10.1016/j.pharmthera.2016.03.013.
